# Screening for asymptomatic mpox in at-risk populations: A cross-sectional study

**DOI:** 10.1371/journal.pone.0335729

**Published:** 2025-10-30

**Authors:** Inés Armenteros-Yeguas, Reynaldo Homen, Adrián Valls, Laura Dans, Eva Orviz, Oskar Ayerdi, Teresa Puerta, Mar Vera, Jorge Alfredo Pérez-García, Montserrat Torres, Mayte Coiras, Jorge Del Romero, Vicente Estrada

**Affiliations:** 1 Centro Sanitario Sandoval, Hospital Universitario Clínico San Carlos, Madrid, Spain; 2 Instituto de Investigación Sanitaria del Hospital Clínico San Carlos (IdISSC), Hospital Clínico San Carlos, Madrid, Spain; 3 PhD Program in Research in Medical-Surgical Sciences, Universidad Complutense de Madrid, Madrid, Spain; 4 Biomedical Research Centre Network in Infectious Diseases (CIBERINFEC), Instituto de Salud Carlos III, Majadahonda, Madrid, Spain; 5 Infectious Diseases Unit, Internal Medicine Service, Hospital Universitario Clínico San Carlos, Madrid, Spain; 6 Immunopathology and Viral Reservoir Unit, National Centre of Microbiology, Instituto de Salud Carlos III, Madrid, Spain; Yamagata University Faculty of Medicine: Yamagata Daigaku Igakubu Daigakuin Igakukei Kenkyuka, JAPAN

## Abstract

**Background:**

Mpox is a viral disease caused by an orthopoxvirus called monkeypox virus. It experienced a significant increase in cases in 2022 worldwide, mostly with sexual transmission. The possibility of hidden circulation of this infection among asymptomatic individuals remains unclear.

**Methods:**

This is a multi-centre, observational cross-sectional study conducted in a sexually transmitted infections (STIs) clinic in its referral hospital between July and October 2023 in Madrid, Spain. Pharyngeal and rectal swab samples were collected from each participant and processed to detect bacterial STIs and mpox. Socio-demographic, clinical and behavioural data were collected, and a descriptive analysis was performed.

**Results:**

A total of 343 asymptomatic participants were included. The prevalence of asymptomatic mpox infection was 0.3% (n = 1) and the only positive case developed symptoms shortly after sampling, ruling out a fully asymptomatic infection. The percentage of vaccinated individuals was 36.1%. 13.2% had previously contracted mpox. Other STIs were detected in 21.6% of participants.

**Conclusions:**

Although routine screening for asymptomatic bacterial sexually transmitted infections is strongly recommended in at-risk individuals, testing for asymptomatic mpox should be evaluated based on the specific context and population. Additionally, the ongoing cases of mpox in Spain are likely not related to the presence of asymptomatic carriers.

## Introduction

Mpox, formerly named monkeypox, is a disease caused by the infection of a DNA virus from the Orthopoxvirus genus within the Poxviridae family [1], described as a cause of disease in humans in 1970 in the Democratic Republic of Congo [2]. It is considered a zoonosis, whose hosts are more probably rodents and mammals that inhabit tropical forests in Africa. However, it could be periodically transmitted to humans through direct contact via bodily fluids, skin lesions, or mucous membranes [3]. Humans could also acquire the virus from person to person through close contact with infectious skin lesions until the lesions crust over. These outbreaks have generally been self-limited, usually confined to the geographical area of central and west Africa, with limited secondary spread among humans [4]. Nonetheless, starting in May 2022, a very high number of cases were described worldwide, in countries where no documented cases had previously existed [5–7], and the World Health Organization (WHO) declared the mpox outbreak a global health emergency [8]. In this outbreak, the predominant pattern of transmission occurred during intimate contact in sexual intercourse, although any close contact can result in transmission [9,10]. Most of the cases have been described in gay, bisexual, or other men who have sex with men (GBMSM) and have presented with a self-limiting clinical course, characterized by different symptoms and signs such as skin lesions, fever, adenopathy, or proctitis [11–14].

Since the start of the 2022 outbreak, Spain has consistently been the third country in the world with the highest number of cases diagnosed, behind the United States and Brazil [5]. The peak incidence coincided with most festive gatherings and overcrowding in the country. Despite later cases decreased, outbreaks and isolated cases continued, so there may be some low-grade transmission in asymptomatic people, as has happened in the past with other viral infections that have led to epidemics [15]. The presence of asymptomatic cases could highlight the variability in how individuals respond to infection according to their immune status [16,17]. Despite the resurgence of the mpox outbreak in Africa in 2024, the hidden circulation of mpox is a question with no answer. Some studies have attempted to address this question; however, they have generally involved small sample sizes and heterogeneous methodologies [18]. New studies with larger cohorts are needed to evaluate the usefulness of screening measures at specific times and among certain at-risk population groups [19,20].

There is data available showing that co-infections of bacterial sexually transmitted infections and mpox have been common, and screening for these infections is recommended in patients with suspected mpox [21–23]. A substantial proportion of these bacterial infections is transmitted by asymptomatic individuals, especially at extragenital sites such as the pharynx and rectum [24]. Consequently, guidelines recommend systematic screening of asymptomatic sexually transmitted infections (STIs) in certain populations [25].

In this study, we determined the prevalence of mpox infection in asymptomatic individuals who attended a STI clinic and its referral hospital. Furthermore, we described the sociodemographic and clinical characteristics of this population, including the prevalence of bacterial STIs, and evaluated the prevalence of mpox vaccination and prior infection status.

## Materials and methods

### Study population and sample collection

This is a multi-centre, observational cross-sectional study conducted in an STI clinic and the Infectious Diseases (ID) unit in its referral hospital in Madrid (Spain) between 17 July and 6 October 2023. The participants were identified when attending a routine screening for STIs at the clinic. The eligible participants were all asymptomatic users with pharyngeal and rectal swabs collected at the same time as they signed the informed consent. We defined asymptomatic people as not having any type of symptoms or signs on physical examination, for mpox or any other STI. Those participants who have suffered a mpox infection within the last six months, or who did not provide informed consent were excluded.

Pharyngeal and rectal swab samples were collected by health workers, by directly taking swabs (eSwab®, Copan, Brescia, Italy) from the mucosa of each site. Some participants were asked to have a urine test for detecting urethral STIs at the election of the clinician. These urine samples were collected by participants in sterile containers and with a system of collection (Vacuette® tube, Greiner Bio-One International, Madrid, Spain) by health professionals.

At the moment of inclusion, the participants completed a questionnaire to collect information on mpox vaccination and number of doses, type of vaccine (mpox or smallpox vaccine) and date of vaccination. Similarly, information on previous mpox and date of the illness was also collected.

Sociodemographic and sexual practices information were obtained from the participant’s medical records and included: gender (cisgender male, transgender male, cisgender female, transgender female or other); date of birth; pre-exposure prophylaxis (PrEP) use and start date; HIV infection and diagnosis date; STIs in the last 6 months; number of sexual partners per month and practices in the last month (use of drugs for sex, group sex, oral sex, vaginal intercourse, insertive anal intercourse and receptive anal intercourse).

### Ethical statement

The study was approved by the Health Care Ethics Committee of the Hospital Clínico San Carlos (23/456-E), following the guidelines of the Declaration of Helsinki. All participants were over 18 years old and gave written consent to be included in the study.

### Description of the laboratory methodology for the detection of mpox and other STIs

Samples were stored and frozen at −20ºC. Pharyngeal rectal and urine samples were processed for simultaneous identification of seven of STIs: *Chlamydia trachomatis* (CT); *Neisseria gonorrhoeae* (NG); *Mycoplasma genitalium* (MG); *Trichomonas vaginalis* (TV); *Mycoplasma hominis* (MH); *Ureaplasma urealyticum* (UU); *Ureaplasma parvum* (UP) using RT-PCR Seegene Allplex® STI Essential Assay Q (MH,UU) (Seegene, South Korea) following the manufacturer’s instructions. Similarly, mpox was detected by RT-PCR employing Novaplex® MPXV Assay (Seegene, South Korea). Cq > 40 were considered negative for Allplex® STI Essential Assay Q and also for Novaplex® MPXV Assay. The nucleic acid extraction was carried out in the automated liquid handling workstation Microlab STARlet-Hamilton (Seegene) and RT-PCR in CFX96 Touch Real-Time (Bio-Rad).

### Statistical analysis

Given the unknown prevalence and the exploratory nature of this study, we determined that screening at least 10 individuals would be necessary to gather meaningful information on potential associations. One previous study by Ferré et al. reported a prevalence of subclinical mpox infection of 6.5% and, according to these data, we assumed a prevalence of 6%, so we would need 100 screenings to find 6 cases and 200 to find 12 [26]. Considering a 6% proportion of asymptomatic subjects in the study population and aiming for a 95% confidence level with a 2.5% margin of error, a sample size of 344 individuals would be required.

Qualitative characteristics of participants were shown using frequency distributions, whereas quantitative variables were reported as means and standard deviations or as medians and interquartile ranges if they did not conform to a normal distribution. Data processing and analysis were conducted using the STATA 15.0 statistical package.

## Results

### Characteristics of participants

A total of 344 participants were included in the study. The power to detect a proportion of 6.5% of our sample was 99%. One participant who subsequently reported symptoms compatible with proctitis at the time of sampling was excluded from the study, leaving a total of 343 participants. The basal characteristics of the participants are shown in Table 1, including potential protective factors such as vaccination and previous mpox immunity due to an earlier mpox infection. Median time since the last dose of vaccine was 320 days (IQR 169-382). In those who had had the disease, the median number of days since diagnosis was 403 days (IQR 367-443).

**Table 1:** Sociodemographic and clinical characteristics of all participants recruited for this study.

Sample size, n	343
**Age, median (IQR)**	36 (30–43)
**Gender**	
Cisgender men, n/N (%)	333/343 (97.1%)
Transgender women, n/N (%)	9/343 (2.6%)
Cisgender women, n/N (%)	1/343 (0.3%)
**Sexual orientation in men**	
Gay, bisexual, or other men who have sex with men (GBMSM), n/N (%)	333/333 (100.0%)
**PrEP use**	
PrEP users, n/N (%)	208/343 (60.6%)
Months using PrEP, median (IQR)	18 (6-31)
**HIV infection**	
People living with HIV (PLHIV), n/N (%)	87/343 (25.4%)
Years since diagnosis of HIV, median (IQR)	8.2 (4.7-13.5)
**Diagnosis of STI in last 6 months, n/N (%)**	194/343 (56.6%)
**Sexual partners *per* month in the previous six months, median (IQR)**	5 (3 –12 )
**Sexual behavioural characteristics of the participants in the last month**	
Oral sex, n (%)	340/343 (99.1%)
Insertive anal sex, n/N (%)	301/343 (87.7%)
Receptive anal sex, n/N (%)	308/343 (89.5%)
Vaginal sex, n/N (%)	17/343 (5.0%)
Use of drugs for sex, n/N (%)	177/343 (51.6%)
Group sex, n/N (%)	66/343 (19.2%)
**Participants with diagnosis of any STI at the time of visit, n/N (%)**	74/343 (21.6%)
**Pharyngeal STI diagnosis, n/N (%)**	23/343 (6.7%)
*Neisseria gonorrhoeae*, n/N (%)	21/343 (6.1%)
*Chlamydia trachomatis*, n/N (%)	2/343 (0.5%)
**Rectal STI diagnosis, n/N (%)**	51/341 (15.0%)
*Neisseria gonorrhoeae*, n/N (%)	32/341 (9.4%)
*Chlamydia trachomatis*, n/N (%)	27/341 (7.9%)
**Urethral STI diagnosis, n/N (%)**	6/122 (4.9%)
*Neisseria gonorrhoeae*, n/N (%)	2/122 (0.6%)
*Chlamydia trachomatis*, n/N (%)	2/122 (0.6%)
*Mycoplasma genitalium*, n/N (%)	4/122 (1.2%)
**Previous vaccination against mpox or human smallpox, n (%)**	124 (36.1%)
Single dose of mpox vaccine, n (%)	50 (14.5%)
Two doses of mpox vaccine, n (%)	60 (17.2%)
Smallpox vaccine in childhood, n (%)	11 (4.4%)
Smallpox vaccine in childhood and one dose of mpox vaccine, n (%)	3 (0.9%)
**Previous infection by mpox, n (%)**	45 (13.2%)
**No previous known infection or vaccination, n (%)**	174 (50.7%)

IQR, interquartile range; PrEP, pre-exposure prophylaxis; STI, sexually transmitted infections.

### Detection of mpox infection

A total of 343 pharyngeal swabs and 341 rectal swabs were processed. Two (0.6%) participants voluntarily requested to participate with only the pharyngeal samples. All samples rendered a valid result on mpox detection by polymerase chain reaction (PCR). Only one individual tested positive for mpox, with both pharyngeal and rectal samples yielding positive results, resulting in a prevalence of 0.3% (1/343). However, 48 hours after inclusion in the study, this individual presented fever and general malaise, with the appearance of skin lesions the following day, reaching a total of 15 lesions distributed on the extremities, face, and trunk. The study samples were collected while he was asymptomatic, but he was probably in the presymptomatic phase of the infection.

This participant was a 42-year-old man born in Spain. His medical history included HIV infection for five years, with antiretroviral treatment and well-controlled immunovirological status (undetectable viral load and CD4 lymphocyte count above 900 cells/uL in the previous year). He had not previously received the mpox or human smallpox vaccine, nor had he had a prior mpox infection. He reported having unprotected oral, insertive anal, and receptive anal sex, but not participating in group sex or using drugs for sex.

Since the prevalence of asymptomatic mpox in our series was low, an analysis of associated factors could not be performed.

### Detection of other sexually transmitted infections

Bacterial STIs are very common in GBMSM, and they are considered biological markers of risk for other infections that must be detected [25]. An asymptomatic screening was performed in this study, finding 74 (21.6%) participants who were diagnosed with at least one asymptomatic bacterial STI (*Neisseria gonorrhoeae*, *Chlamydia trachomatis* or *Mycoplasma genitalium* infection) in the pharynx, rectum, or urethra. The prevalence of STIs is listed in Table 1.

Rectal infections were the most common (15.0%), followed by pharyngeal (6.7%) and urethral sites (4.9%). *Neisseria gonorrhoeae* was the most frequently detected microorganism, with 32 rectal samples (9.4%), 21 pharyngeal samples (6.1%), and 2 urine samples (0.6%). *Chlamydia trachomatis* was primarily detected in rectal samples (27, 7.9%), with low detection values at other sites (2 pharyngeal samples, 0.6%; 2 urine samples, 0.5%).

The participant who tested positive for mpox in pharyngeal and rectal swabs was also found to have a positive urine test for *Neisseria gonorrhoeae*.

## Discussion

This observational, cross-sectional study described the prevalence of asymptomatic mpox in a population at risk for STIs in Spain, which is the third country in the world with the highest number of mpox cases diagnosed so far [5]. The study setting is an STI clinic and the follow-up unit of people living with HIV in the partner hospital. A sample of nearly 350 participants was recruited, making this one of the largest mpox screening studies published in the literature to date.

We aimed to identify mpox healthy carriers because these individuals can transmit pathogens without showing any symptoms, essentially acting as reservoirs of infection. Even in these subjects, pathogens can evolve or adapt, leading to the emergence of new strains or variants that may be more transmissible, virulent, or resistant to treatment, which may complicate disease management further.

The prevalence of mpox found in this study was 0.3%, lower than initially expected (6.5% in the study by Ferré et al. [26]) or identified in previous studies with fewer participants recruited (1% in a Swiss cohort of 201 participants [27]).

Additionally, the participant with positive pharyngeal and rectal samples developed mucocutaneous lesions 48 hours after inclusion in the study, indicating that he did not have an asymptomatic infection, which was the primary focus of this investigation. This information was consistent with previously published literature in which a search for mpox was performed exclusively in asymptomatic subjects, revealing an absence of mpox subclinical circulation [28]. Other studies that searched for subclinical mpox found higher prevalences (6.19% [29]; 1.78% [30]). However, these studies included people without symptoms associated with mpox, but they might have had other symptoms at the time of sampling. One Japanese study by Mizushima et al. with a large cohort (1,346 participants) reported an asymptomatic mpox prevalence of 0.29%, which is very similar to the findings of our study [31].

In this study, mpox PCR tests were positive at extragenital sites before the disease’s defining symptoms appeared, similar to one case in the Mizushima et al. study, where symptoms developed three days after testing. This suggests there is an asymptomatic period before symptoms, during which the individual could transmit the infection. It may be useful for reinforcing preventive behaviours in asymptomatic contacts of infected persons on days of the incubation period [32]. Nevertheless, our findings indicate that this does not seem to be the primary mechanism of transmission.

The absence of asymptomatic mpox in the studied population is not due to a selection bias towards a low-risk population; rather, the proportion of STIs is higher than that reported in other studies about STI screening in asymptomatic subjects (19.9% [33], 14.6% [34], and between 16.2% and 22.1% [35]).

Another possible explanation for the absence of cases in our series could be the low incidence of the disease in our region during the study period. In Spain, after the 2022 epidemic, mpox cases have appeared intermittently. However, during the study recruitment period, a total of 66 cases were reported, with 14 out of 66 (21.2%) diagnosed in our clinic during that time. This indicates that active transmission was occurring [36].

We have not identified any asymptomatic mpox carriers in our extensive series of individuals studied, despite having a high percentage of STIs as well as having other mpox cases diagnosed in our centre. This finding suggests that the healthy mpox carrier state is rare and does not explain the sporadic cases observed in the months following the 2022 outbreak. The emergence of new cases in populations with inadequate immune responses to this pathogen, driven by significant human mobility in a globalized world, could be the cause for the cases noted. This is consistent with previous data on genomic variability between cases diagnosed in the same country [37]. Therefore, it is advisable to strengthen preventive measures and vaccination efforts among potentially vulnerable people.

This study presented several limitations. First, the participants were individuals who sought screening tests through the National Health System (NHS), which may introduce selection bias concerning those who do not usually utilize the NHS and could be more susceptible to STIs. Second, the study was conducted in a centre specializing in infectious diseases, which may also introduce a selection bias regarding persons evaluated at non-specialized centres. Consequently, the population included in the study consisted only of a specific group of subjects and might not represent the entire population at risk. Another limitation to consider is that only a single sample was collected from each participant, so infections that may have developed later could not be excluded, given the incubation period of up to 21 days. Moreover, this study was conducted under specific epidemiological circumstances, with a low incidence of cases, so the results may not be extrapolated to other epidemiological settings with a higher incidence rate. Finally, having only one case prevented us from analyzing associated factors, representing a study limitation.

The strengths of our study include a large number of patients, making it one of the most extensive series of mpox screening in asymptomatic subjects reported to date, along with comprehensive clinical information collected.

## Conclusions

It is suggested that mpox Clade IIb may manifest with no symptoms or only mild symptoms [38,39]; however, the prevalence of asymptomatic mpox in the population studied, considered at risk for STIs, was almost null. Testing for various asymptomatic STIs is recommended in many situations, while testing for asymptomatic mpox may differ depending on location and population. There is insufficient evidence to confirm that ongoing cases of mpox in Spain are attributable to asymptomatic carriers of the infection.
